# Prevalence of Vitamin D Deficiency in Patients Treated for Juvenile Idiopathic Arthritis and Potential Role of Methotrexate: A Preliminary Study

**DOI:** 10.3390/nu14081645

**Published:** 2022-04-14

**Authors:** Maciej K. Stawicki, Paweł Abramowicz, Adrian Góralczyk, Justyna Młyńczyk, Anna Kondratiuk, Jerzy Konstantynowicz

**Affiliations:** 1 Department of Pediatrics, Rheumatology, Immunology, and Metabolic Bone Diseases, Medical University of Bialystok, Waszyngtona Street 17, 15274 Bialystok, Poland; maciej.stawicki@umb.edu.pl (M.K.S.); justyna.mlynczyk@umb.edu.pl (J.M.); an.kondratiuk@gmail.com (A.K.); jurekonstant@o2.pl (J.K.); 2 Department of Orthopaedics and Traumatology, Hospital of Ministry of Administration and Internal Affairs in Bialystok, Fabryczna Street 27, 15471 Bialystok, Poland; adriangoralczyk1@yahoo.com

**Keywords:** juvenile idiopathic arthritis, vitamin D, calcium/phosphate metabolism, methotrexate, children

## Abstract

Background: Vitamin D deficiency is reported in rheumatological diseases in adults. The aim was to evaluate the prevalence of vitamin D deficiency in children with juvenile idiopathic arthritis (JIA) and to investigate potential correlations between vitamin D status and clinical factors, laboratory traits, and medical treatment, including methotrexate (MTX) and glucocorticoids (GCs). Methods: In 189 patients aged 3–17.7 years, with JIA in the stable stage of the disease, anthropometry, clinical status, serum 25-hydroxyvitamin D [25(OH)D], calcium (Ca), phosphate (PO_4_), total alkaline phosphatase (ALP), erythrocyte sedimentation rate (ESR), and C-reactive protein (CRP) were assessed. Results: Median 25(OH)D level was 15.00 ng/mL, interquartile range (IQR) 12.00 ng/mL. Vitamin D deficiency was found in 67.2% and was independent of sex, disease manifestation, and CRP, ESR, ALP, or PO_4_ levels. Higher doses of MTX corresponded with lower 25(OH)D levels using both univariate and multivariate models (*p* < 0.05). No such trend was found for GCs treatment. Serum Ca was lower in patients treated with GCs (*p* = 0.004), MTX (*p* = 0.03), and combined GCs/MTX (*p* = 0.034). Conclusions: JIA patients are vitamin D depleted independently of disease activity or inflammatory markers. MTX therapy may be an iatrogenic factor leading to inadequate 25(OH)D levels. Vitamin D supplementation should be considered in all children with JIA, particularly those receiving long-term MTX therapy.

## 1. Introduction

Juvenile idiopathic arthritis (JIA) is a heterogenic group of chronic autoimmune disorders with a large spectrum of clinical manifestations and varying severity [1]. The causes of the disease are yet to be discovered. It is suggested that immunogenic mechanisms secondary to genetic and environmental factors are the background of the disease, while infections together with stress and trauma are suspected to be the most possible etiological agents for JIA [2].

As the incidence of JIA has been reported to increase worldwide, the management of this condition has become an important issue in pediatric care [3,4]. The complex autoimmune, inflammatory, and destructive processes, which are key pathogenic mechanisms in the disease, may lead to disability early in childhood and adolescence and may either persist to adulthood or confer a risk of later significant rheumatologic conditions including rheumatoid arthritis (RA).

Vitamin D is a fat-soluble vitamin and an important hormone involved in many physiological processes in the human body, such as bone mineralization, insulin regulation, and immune regulation [5,6,7]. Vitamin D is affecting the tissues by specific vitamin D receptors (VDR) inducing its biological activities. VDR is widely expressed in different cells, i.e., immune cells. There are many polymorphic variants of the VDR gene that possibly affect the functionality of the receptor [8].

Recent research focused on vitamin D in relation to a variety of inflammatory disorders revealed several controversial results. Furthermore, a large body of evidence was published within the last decade to demonstrate a vast range of vitamin D deficits in general, otherwise healthy, populations worldwide [9,10,11,12,13,14]. The role of vitamin D, being mainly a well-known regulator of calcium/phosphate metabolism, has been extensively investigated in adult rheumatoid diseases, demonstrating a potential beneficial effect on the disease course and activity; however, some studies did not support such associations [15].

Available reports show that vitamin D may have a significant influence on pathogenesis [16] and the outcome of JIA, i.e., a lower level of 25-hydroxyvitamin D [25(OH)D] was found in JIA patients compared with healthy children [17,18]. At present, there is a strongly held general view, based also on prospective studies, that vitamin D has pleiotropic multidimensional effects on human metabolism and may interact in situ with specific tissues and, therefore, demonstrates some preventive potential including anti-proliferative, anti-inflammatory, and immunomodulatory actions. On the other hand, the long-lasting vitamin D deficiency, reflected by decreased 25(OH)D concentrations, can deteriorate immune-mediated mechanisms or even exacerbate the course of the disease [19].

Assuming that vitamin D in pediatric rheumatoid diseases may be of importance and that several questions regarding vitamin D deficit remain unanswered, we attempted to investigate correlations between vitamin D status, clinical manifestation, and medical treatment of JIA. This study aimed to determine the prevalence of vitamin D deficiency and to evaluate potential risk factors of decreased serum 25-hydroxyvitamin D levels in children diagnosed with JIA.

## 2. Material and Methods

In this cross-sectional study, 189 Caucasian individuals (both in- and outpatients) treated for juvenile idiopathic arthritis were examined. The diagnosis of JIA was ascertained using standard classification criteria [20]. Clinical assessment, anthropometric measurements using standardized methods, and laboratory tests were performed. Blood samples were collected at the beginning of hospitalization. Clinical assessment, based on physical examination and functional tests, was performed during scheduled hospital admission. Juvenile arthritis disease activity score (JADAS27) was used to determine disease activity status [21]. Anthropometric measurements were carried out with standardized methods, compliant with WHO guidelines [22], and included body weight using an electronic scale (Seca^TM^ 799, Hamburg, Germany) and standing height obtained with a wall-mounted stadiometer (Seca^TM^ 216, Hamburg, Germany). Body Mass Index (BMI) was calculated with a standard formula.

Vitamin D status was determined by measuring serum 25(OH)D concentration using the automatic immunoenzyme method using Immulite^®^2000 Immunoassay System (Siemens AG, Munich, Germany). Vitamin D deficiency was defined as serum 25(OH)D level < 20 ng/mL, consistently with the Institute of Medicine recommendations and the updated guidelines for Central Europe [5,10]. To assess inflammation activity, erythrocyte sedimentation rate (ESR), and C-reactive protein (CRP) concentration were measured. Calcium (Ca) and phosphate (PO_4_) serum concentrations and total alkaline phosphatase (ALP) activity were also tested and referred to age-specific normative values to check basic bone mineral metabolism.

All procedures were approved by the Ethical Committee of the Medical University of Bialystok upon informed consent obtained from all participants and/or their legal guardians according to the Declaration of Helsinki and its later amendments.

The statistical analyses were performed with the STATISTICA software (version 13.3, Tibco Software Inc., Palo Alto, CA, USA) and statsmodels.org (version 0.13.2). To evaluate the normality of data distribution, Shapiro–Wilk test was used. Variables distributed normally were expressed as mean and standard deviation, whereas for those with skewed distribution, median and IQR were used as a method of result presentation. According to the data distribution, Student’s *t*-test or, the Mann–Whitney *U* test was applied. Subsequently, Spearman rank correlation was used to test the relation between pairs of factors. Furthermore, multinomial logistic regression was used to investigate associations between 25(OH)D concentration and body weight, BMI, MTX dose, GCs dose, CRP, ESR, Ca, P, and ALP, which were incorporated as covariates in the models.

## 3. Results

A total of 189 children and adolescents (113 girls and 76 boys), aged 3–17.7 years (median 13.12, IQR 6.23) were included; all were Caucasian, living at a similar latitude, none of the participants had been diagnosed with comorbidities potentially affecting vitamin D or bone metabolism, and none had been supplemented with vitamin D at the time of the recruitment to the study.

Among the whole studied group, 49% had oligoarticular manifestation, 44% presented polyarticular manifestation, and 7% had systemic-onset JIA (Table 1)**.** All of them were in a stable stage of the disease (remission or minimal disease activity) according to the JADAS27 scoring [21].

Methotrexate at a weekly dose of 10–20 mg per m^2^, administered orally or subcutaneously, was the only conventional synthetic disease-modifying anti-rheumatic drug (csDMARD) used in these patients, whereas no other DMARDs combination was introduced. In addition, some patients required interim GCs as a bridging therapy, in rare cases given by intra-articular injections.

The median 25(OH)D serum concentration was 15.00 ng/mL, and the IQR was 12.00 in the whole studied group. Vitamin D deficiency was found in 127 patients of both sexes (67.2% of the examined population). Comparisons between the groups in relation to serum 25(OH)D concentration based on the cut-off level of 20 ng/mL are shown in Table 2.

Vitamin D status in children with JIA was independent of sex, age, clinical manifestation, disease activity, or inflammatory markers. Serum 25(OH)D was inversely associated with BMI (r = −0.19), i.e., overweight JIA patients had lower vitamin D levels. Additionally, a weak yet significant correlation between 25(OH)D and serum Ca (r = 0.19) was found. An association was observed between vitamin D status and pharmacological therapy used in children with JIA. Furthermore, the treatment option affected calcium/phosphate metabolism in both boys and girls. Weekly MTX dose was found to be significantly higher in patients with vitamin D deficiency than in those with serum 25(OH)D > 20 ng/mL (Table 2). This association was consistent with Pearson’s correlation coefficient, indicating an inverse relationship between the dose of MTX and 25(OH)D concentration (r = −0.34, *p* < 0.05). There was a significant negative correlation between MTX dose and Ca and PO_4_ levels but not with serum ALP. Furthermore, the GCs dose had no significant effect on serum 25(OH)D concentration in children with JIA, whereas the daily dose of GCs was inversely associated with ALP activity, Ca, and PO_4_ levels. Significant correlations between the two treatment options are shown in Table 3.

Multinomial logistic regression analysis showed that, out of all factors introduced in the model, only the weekly MTX dose per m^2^ was inversely associated with serum 25(OH)D concentration. The calculated coefficient was 1.79 for MTX/week/m^2^ (95% confidence interval [CI]: 0.33–3.24), whereas no other variables were significantly associated with vitamin D concentration. The results of the multivariate analysis are presented in Table 4.

## 4. Discussion

Vitamin D deficiency is common in the general population during growth, according to the available supportive evidence [9,10,11]. This observation has been extended by the present study demonstrating a disease-specific deficiency in patients with juvenile idiopathic arthritis. Several studies have reported suboptimal vitamin D status in children with rheumatic diseases resulting from multifactorial mechanisms associated with the autoimmunity and/or iatrogenic effects [4,15]. The main finding of our study was an association between long-term methotrexate therapy in children with JIA and a deteriorated vitamin D status. Due to the cross-sectional design of this study, causal pathways may not be clearly elucidated; however, the unfavorable effect of the MTX therapy on 25-hydroxyvitamin D may indicate a role of this particular medication in an increased risk of deficiency.

Presumably, the above-mentioned treatment essentially affected the vitamin D status and calcium/phosphate metabolism, as it was found to interfere at most with vitamin D deficiency, among other variables analyzed in this study. Noteworthy, the strategies of therapeutic management are similar in RA and JIA, excluding current therapies with a single drug, e.g., biologics. Effective recommendations include the proposal of subsuming a sequential application of non-steroidal anti-inflammatory drugs (NSAIDs), GCs, and non-biological/biological DMARDs in the treatment of RA depending on disease activity and severity [23]. Similarly, the currently binding approach to the complex medication of children with juvenile arthritis is based on analogous recommendations [24]. Methotrexate—out of all non-biological DMARDs—appears the most effective and preferably applicable agent due to its well-known effectiveness for restricting autoimmune and inflammatory processes. Moreover, recent studies have shown that MTX is also an inhibitor of osteoclastogenesis by impeding RANKL-induced calcium influx into osteoclast progenitor cells [25]. Kanagawa et al. postulated that MTX would have a protective role against osteoporosis and joint destruction via some of the above-mentioned specific mechanisms [25]. In the light of the multivariate approach, the results of our study suggest a different view, showing that MTX use may be associated with a decreased 25(OH)D level. Initial univariate analyses also showed a dose-dependent effect, i.e., the weekly dose of MTX is negatively associated with serum calcium and phosphate, although multivariate analyses failed to support those results. The influence of MTX on calcium/phosphate metabolism can be direct or indirect—just by affecting vitamin D metabolism. Assuming these causal effects may be true, a question arises: During which transformation phase of vitamin D precursors does this drug interfere? Possible interaction may occur at intestinal absorption, during which methotrexate may deteriorate vitamin D bioavailability from nutrients. Furthermore, it can also considerably downregulate hepatic hydroxylation of calciferol (due to its fully understood liver toxicity), or it can even affect skin synthesis of vitamin D.

These associations have not yet been probably reported in the literature concerning rheumatoid diseases, i.e., JIA or RA. Methotrexate may be regarded as a risk factor for secondary osteoporosis in adults, even if the available data are inconsistent [26,27]. Nevertheless, there is a need for further relevant investigation to determine if long-term MTX use is an independent factor of bone loss in children with rheumatoid conditions. Moreover, a workout of a molecular mechanism through which MTX affects vitamin D metabolism is necessary to prevent the negative effects of MTX treatment on the growing skeleton.

Surprisingly, the prolonged use of GCs in our patients was not associated with a decrease in 25(OH)D concentration despite an evident inverse relationship between GCs and calcium or phosphate metabolism. Some studies support our results by demonstrating clearly that the use of systemic steroids does not influence 25(OH)D levels [28]. Other reports show, by contrast, that GCs may have a specific regulatory effect on vitamin D metabolism [19,29]. Our finding seems even more interesting considering an insight into the molecular mechanism of actions of GCs, reflecting “anti-vitamin D effects”. Possible explanations include that glucocorticoids increase calcium and phosphate renal excretion, being antagonists of 1,25(OH)_2_D, while not influencing its serum concentration. Furthermore, there was a strong negative association between the GCs dose and total alkaline phosphatase activity. It has been reported that the decreased serum Ca and PO_4_ levels, as well as reduced ALP activity, may be a compelling contribution to reduced bone mineral apparent density (BMAD) concomitant with long-term GCs treatment in children with JIA [30]. The causal relationship between exposure to GCs and suboptimal bone mineral acquisition during growth, including glucocorticoid-induced osteoporosis, has been widely documented, although not all mechanisms have been clarified.

Disease activity, duration, and active inflammation play an important role in the deterioration of mineral and bone metabolism in the course of chronic rheumatologic conditions [15]. We point out that the inflammatory process is supposed to be another risk factor for vitamin D deficiency in children with confirmed JIA. Several published reports are attempting to elucidate this association, but the results are inconsistent and provide ambiguous information [31]. To optimize the usefulness of our study, the JADAS27 scale was applied for disease activity assessment. All studied patients were in remission or had minimal activity of JIA based on the JADAS criteria [21]. These characteristics allowed us to minimize the effect of the confounder, i.e., the impact of disease activity and severity on the results. Based on large cohort studies, there was an inverse relationship between vitamin D intake and RA disease risk [32]. However, the data are different when comparing RA and JIA, while results vary across published studies. More recent reports showed that vitamin D level was significantly reduced in patients with active RA [33,34,35,36]. In those studies, the scores DAS28, JADAS-27, and inflammatory markers (ESR, CRP, fibrinogen serum concentration) were used to assess disease activity. Some investigators reported that the prevalence of vitamin D deficiency or insufficiency was high in juvenile idiopathic arthritis; however, it was unassociated with either intensity of inflammation [37,38,39] or the genotypes of the vitamin D receptor [8]. Other studies revealed the relationship between the inflammatory process and reduced 25(OH)D [40,41,42]. Active forms of vitamin D have been shown to diminish the inflammatory process through the inhibition of interleukin-6 which is a key cytokine involved in joint destruction [43]. Finally, more profound and corrected analyses may detect true associations. There are published studies in which univariate analyses indicating significant correlation have been essentially altered by multivariate analyses [44]. Our study supports the view that 25(OH)D is independent of disease activity, despite a slight negative correlation between ESR and vitamin D levels. We believe that inflammatory markers alone may not be specific enough to assess disease activity categorically, as both clinical manifestation and the severity of JIA are determined by a multiplicity of factors. If so, it was difficult to find or confirm associations between biochemical markers of inflammation and 25(OH)D serum concentration in this study. Some reports showed an association between a clinical manifestation of RA or JIA and the overall disturbance of the metabolism [45]. Interestingly, in the present study, no differences were observed in vitamin D status between individuals with polyarthritis and systemic-onset JIA even after adjustment for age and sex as possible confounding effects.

In summary, our preliminary study showed that methotrexate may have a general negative influence on vitamin D status in children with JIA. This confers a possible risk of deteriorated bone density and impaired skeletal accrual during growth. There is always a need for supplementation of vitamin D and calcium to be considered in these patients accordingly to the general guidelines. The maintenance of the optimal vitamin D status can be useful in reducing pain symptoms and improving the quality of life in these children, as it was proven in patients with RA [46], considering particular pleiotropic effects of calcitriol, including anti-inflammatory, immunomodulatory, and cell-protecting features. Though there are still some controversial findings in the literature suggesting that vitamin D supply, although effective, does not sufficiently enhance bone health. For example, Hillman et al. proved that vitamin D_3_ treatment with 2000 IU/day plus calcium increased 25(OH)D concentration and allowed maintaining the 1,25(OH)_2_D level but did not improve BMD accretion [47].

We are aware that our study has some relevant limitations, and the cross-sectional design does not allow establishing firm conclusions concerning causal effects. Accessibility to relevant solid data was limited, specifically related to the duration of GCs, and MTX therapy was ineffective. The difficulty resulted from the study design, as the treatment courses were established individually for each patient and were subsequently adjusted depending on clinical manifestation and course of the disease. Because of essential technical issues, it was not possible to assess skeletal status by measuring bone mineral density.

## 5. Conclusions

The majority of children with juvenile idiopathic arthritis have significantly decreased 25-hydroxyvitamin D serum concentrations independent of clinical manifestations, disease activity, age, sex, or inflammatory markers. Iatrogenic factors play an important role in the development of vitamin D deficiency in JIA. According to our study, long-term methotrexate therapy appears to be the factor associated with reduced 25(OH)D levels. Although glucocorticoids used in JIA essentially affect calcium/phosphate metabolism, indicators of their influence on vitamin D status were not found. Our findings suggest the necessity of extensive vitamin D supplementation in children with JIA, particularly those treated with methotrexate. There is a need for further studies on the effects of methotrexate on vitamin D status in this population with special regard to the underlying molecular mechanism.

## Figures and Tables

**Table 1:** Basic characteristics of the study group (* Median and IQR value are shown when applicable).

	Total (*n* = 189)
Age (years) *	13.12 (6.23)
Male-to-female	76/113
Weight (kg) *	48.50 (24.00)
Height (cm) *	155.00 (28.00)
BMI (kg/m^2^) *	19.58 (5.26)
Polyarticular JIA (*n*; %)	83 (43.90%)
Oligoarticular JIA (*n*; %)	93 (49.20%)
Systemic-onset JIA (*n*; %)	13 (6.90%)
Treated with GCs (*n*; %)	73 (38.60%)
Treated with MTX (*n*; %)	84 (44.40%)

**Table 2:** Characteristics of the patients with JIA in relation to their serum 25(OH)D concentration below and above 20 ng/mL (* mean ± SD or ** median and IQR are given).

	Low 25(OH)D Level <20 ng/mL	Normal 25(OH)D Level ≥20 ng/mL	*p* Value
Patients (N; %)	127 (67.2%)	62 (32.8%)	
Age (years) **	13.34; (5.18)	11.84 (8.32)	0.19
Weight (kg) **	50.00 (22.00)	45.35 (33.00)	0.14
Height (kg) **	156.50 (25.00)	148.00 (37.50)	0.15
BMI (kg/m^2^) **	19.81 (4.88)	19.06 (6.10)	0.17
GCs (mg) ** daily dose	5.00 (5.00)	5.00 (5.00)	0.29
MTX (mg) ** weekly dose per m^2^	15.00 (7.50)	12.5 (7.50)	0.02
CRP (mg/L) **	1.00 (3.70)	1.60 (12.0)	0.23
25(OH)D (ng/mL) **	12.00 (8.00)	25.5 (6.0)	<0.001
Ca (mmol/L) *	2.48 ± 0.09	2.52 ± 0.12	0.01
P (mg/dL) *	4.50 ± 0.63	4.54 ± 0.72	0.76
ALP (U/L) **	165.00 (138.00)	172.00 (124.00)	0.70
ESR (mm/h) **	11.50 (22.00)	19.00 (30.00)	0.05
JADAS27 score **	1.50 (0.70)	1.50 (0.50)	0.64

**Table 3:** Univariate correlations for methotrexate (MTX), glucocorticoids (GCs), and serum calcium/phosphate parameters in children with JIA.

	MTX *weekly dose per m*^2^	GCs *daily dose*
25(OH)D	r = −0.33; *p* = 0.003	r = −0.08; *p* = 0.26
Calcium	r = −0.31; *p* = 0.01	r = −0.23; *p* = 0.01
Phosphate	r = −0.42; *p* = 0.03	r = −0.27; *p* = 0.004
ALP	r = −0.14; *p* = 0.17	r = −0.79; *p* = 0.004

**Table 4:** Results of multinomial logistic regression performed to investigate multivariate analysis of factors associated with 25(OH)D concentration.

	Coefficient	Standard Error	95% CI		*p* Value
Body weight	0.09	0.40	−0.69	0.87	0.82
BMI	−0.08	0.32	−0.70	0.54	0.80
GCs *daily dose*	0.18	0.21	−0.24	0.60	0.41
MTX *weekly dose per m^2^*	1.79	0.74	0.33	3.24	0.02
CRP (mg/L)	−0.29	0.23	−0.74	0.17	0.21
ESR (mm/h)	−0.72	0.23	−0.51	0.37	0.75
Ca (mmol/L)	−0.13	0.19	−0.51	0.24	0.48
P (mg/dL)	0.32	0.21	−0.10	0.73	0.13
ALP (U/L)	0.01	0.20	−0.37	0.40	0.94

## Data Availability

Data are available on request.
